# PGE2 Supplementation of Oocyte Culture Media Improves the Developmental and Cryotolerance Performance of Bovine Blastocysts Derived From a Serum-Free *in vitro* Production System, Mirroring the Inner Cell Mass Transcriptome

**DOI:** 10.3389/fcell.2021.672948

**Published:** 2021-06-07

**Authors:** Gilles Charpigny, Brigitte Marquant-Le Guienne, Christophe Richard, Pierre Adenot, Olivier Dubois, Valérie Gélin, Nathalie Peynot, Nathalie Daniel, Vincent Brochard, Fabienne Nuttinck

**Affiliations:** ^1^UVSQ, INRAE, BREED, Université Paris-Saclay, Jouy-en-Josas, France; ^2^Ecole Nationale Vétérinaire d’Alfort, BREED, Maisons-Alfort, France; ^3^ALLICE, BREED, Jouy-en-Josas, France; ^4^INRAE, MIMA2, Université Paris-Saclay, Jouy-en-Josas, France

**Keywords:** periconceptional environment, prostaglandin signaling, embryo development, apoptosis, cell cycle, embryonic cell, pluripotency, epigenetic regulation

## Abstract

The culture media used throughout the *in vitro* production (IVP) of bovine embryos remain complex. The serum added to culture media in order to improve embryo development negatively impacts the cryotolerance of blastocysts. Periconceptional prostaglandin E2 (PGE2) signaling is known to exert prosurvival effects on *in vitro*-generated blastocysts. The purpose of the present study was to evaluate the effects on developmental and cryotolerance performance of a serum-free (SF) IVP system that included defined oocyte culture media supplemented or not with PGE2, versus serum-containing (SC) IVP. RNA-sequencing analysis was used to examine the gene expression of ICM derived under the different IVP conditions. We assessed the degree of cryotolerance of grade-I blastocysts during a three-day post-thaw culture by measuring survival and hatching rates, counting trophectoderm and inner cell mass (ICM) blastomere numbers. We also determined the proportion of ICM cells expressing octamer-binding transcription factor 4 protein (OCT4/POU5F1). We showed that grade-I blastocyst development rates under SF + PGE2 conditions were similar to those obtained under SC conditions, although the cleavage rate remained significantly lower. SC IVP conditions induced changes to ICM gene expression relative to several metabolic processes, catabolic activities, cell death and apoptosis. These alterations were associated with significantly higher levels of ICM cell death at day 7 post-fertilization, and lower survival and hatching rates after thawing. SF IVP conditions supplemented or not with PGE2 induced changes to ICM gene expression related to DNA replication, metabolism and double-strand break repair processes, and were associated with significantly larger ICM cell populations after thawing. SF + PGE2 IVP induced changes to ICM gene expression related to epigenetic regulation and were associated with a significantly higher proportion of ICM cells expressing OCT4. For the first time, our study thus offers a comprehensive analysis of the ICM transcriptome regulated by IVP culture conditions in terms of the cellular changes revealed during culture for three days after thawing.

## Introduction

The cryopreservation of embryos is used routinely by cattle breeders as well as in the field of human assisted reproductive technology (ART) (Maheshwari et al., 2018; Zhengyuan et al., 2019; Bosch et al., 2020). In cattle, embryo freezing contributes to improving the management of recipient cows as well as commercial worldwide exchanges within the livestock industry. Associated with biopsy and genotyping, it can accelerate genetic improvement through breeding programs. In addition, measuring embryo survival after cryopreservation may offer an approach to assess the quality of embryos produced *in vitro* (Carrocera et al., 2016). In humans, embryo cryopreservation enables the postponement of pregnancy relative to the time of fertilization, offering an opportunity to preserve female fertility over time, and even after cytotoxic anticancer therapy, for example. It can also improve the management of supernumerary embryos following ovarian stimulation.

However, freezing damages cells and may adversely affect embryo survival. The cryotolerance of embryos is influenced by a wide range of factors related not only to the freezing technique but also to the embryo itself (Li et al., 2014; Gupta et al., 2016; Mandawala et al., 2016; Rienzi et al., 2017; Cai et al., 2018). The source of the embryo may affect its ability to withstand freezing (Polge and Willadsen, 1978; Alviggi et al., 2018). Studies performed in cattle have shown that embryos produced *in vitro* are more vulnerable to cooling than their counterparts generated *in vivo* (Nedambale et al., 2004; Yu et al., 2010). In addition to the absence of a natural environment for oocytes and embryos, the composition of the culture media used throughout the successive steps of *in vitro* production (IVP); namely *in vitro* maturation (IVM), *in vitro* fertilization (IVF), and *in vitro* development (IVD), may modulate embryo survival after thawing (Agarwal et al., 2014; Swain et al., 2016; Ferre et al., 2020). In cattle, it has been well known for decades that adding serum to culture media negatively affects embryo cryotolerance (Yamashita et al., 1999). A reduction in post-thaw viability in response to serum-supplemented IVP conditions has been thought to result from changes to energy metabolism and an accumulation of intracellular lipids in embryonic cells (Khurana and Niemann, 2000; Abe et al., 2002; Reis et al., 2003; George et al., 2008). Several transcriptomic studies have highlighted the impacts of the IVP procedure itself, as well as the composition of the culture media used throughout IVP (and particularly, the presence of serum), on global transcriptomic profiles at the blastocyst stage. When compared with *in vivo* embryos, analysis of the transcription status of *in vitro* blastocysts gives an overall picture of how the embryo responds to its different environments (Hosseini et al., 2015; Cagnone and Sirard, 2016). The IVP procedure has been shown to induce stress responses in the embryo, reflected by changes to the expression of pathways related to energy metabolism, extracellular matrix remodeling and inflammatory signaling. When compared with an IVP system totally devoid of hormones and serum, it was reported that the serum supplementation of culture media had a major impact on the global gene expression pattern of *in vitro* bovine blastocysts, and particularly of genes related to lipid metabolism and DNA repair processes (Heras et al., 2016). These authors also observed that the transcriptome of embryos generated *in vitro* was more similar to that of *in vivo* counterparts when IVP was performed in the absence of serum.

Previous studies performed in cattle showed that embryonic apoptosis was amplified in response to the serum supplementation of IVD cultures (Byrne et al., 1999; Rizos et al., 2003; Sudano et al., 2011). A serum-induced increase in apoptosis was evidenced in both fresh and thawed blastocysts, and was associated with reduced cryotolerance (Sudano et al., 2011). Embryonic apoptosis is a physiological activity that indicates the developmentally controlled elimination of cells, and it affects preimplantation embryos in many mammals (Fabian et al., 2005). In cattle, a wave of embryonic cell death occurs during development of the non-expanded to the hatched blastocyst, and mainly affects the inner cell mass (ICM) (Gjorret et al., 2003, 2007). We have previously shown that the degree of ICM cell death may have an impact on subsequent development. Weaker apoptotic activities at the blastocyst stage were associated with more advanced post-hatching development after fresh embryo transfers and the highest proportions of conceptuses in which the antero-posterior patterning of the embryonic disk was initiated two weeks after IVF (Nuttinck et al., 2017). An earlier study in humans that combined mathematical modeling and experimental *in vitro* observations suggested that the factors predisposing an embryo to develop normally or to arrest are largely determined at the one-cell stage (Hardy et al., 2001). Using a serum-free IVP system, we demonstrated that the prostaglandin E2 (PGE2) enrichment of bovine oocyte culture media conferred increased resistance to spontaneous embryonic apoptosis at the blastocyst stage (Nuttinck et al., 2017). PGE2 is an arachidonic acid-derived lipid mediator present in the natural environment of the oocyte during the periconceptional period in several mammalian species (Nuttinck et al., 2002; Duffy et al., 2019; Boruszewska et al., 2020; Rouhollahi Varnosfaderani et al., 2020). In response to the gonadotropin surge, mural granulosa and cumulus cells produce PGE2, whose levels in the follicular fluid rise to micromolar concentrations prior to ovulation (Duffy, 2015). Increasing the PGE2 concentration in the oocyte culture medium to a level similar to that observed in the follicular fluid was designed to mimic the *in vivo* situation. In addition to its prosurvival effect on embryonic cells, the presence of PGE2 in the oocyte environment promoted cell cycle kinetics throughout *in vitro* development, leading to an increased number of total blastomeres per embryo (Nuttinck et al., 2011; Nuttinck et al., 2017; Rouhollahi Varnosfaderani et al., 2020). But although the involvement of periconceptional PGE2 in controlling bovine embryonic cell populations has been established, its beneficial effects on thawing survival still need to be explored.

Attempts have been made over several years to reduce the amount of serum present in the culture media used during IVP in cattle (Block et al., 2010; Zolini et al., 2020). Nevertheless, IVP culture media are still supplemented with serum because this may enable higher percentages of zygotes to reach the blastocyst stage, i.e. the developmental stage required for embryo transfer (George et al., 2008; Sudano et al., 2011). PGE2 is present at nanomolar levels in standard serum-containing maturation media (unpublished data). In any case, the composition of commonly employed oocyte culture media remains complex because as well as serum, they may also be supplemented with different hormones such as follicle stimulating hormone, luteotropin hormone and 17β-estradiol. However, the impact of IVP conditions on specific biological processes that occur within the ICM during establishment of the first cell lineages remains unclear. The objective of the present work was therefore to explore at the cellular and molecular levels the effects on blastocyst development and cryotolerance of a simple and defined oocyte culture medium, devoid of hormones and enriched or not with PGE2, combined with serum suppression throughout the IVP procedure. According to our previous findings (Nuttinck et al., 2011, 2017), the optimal PGE2 concentration of 1 μM was added to the IVM and IVF media. The cryotolerance of blastocysts was investigated after conventional slow freezing by measuring their survival rate throughout a three-day post-thaw culture. In addition, the ICM quality of day 3 post-thaw blastocysts was evaluated by (i) counting ICM and trophectoderm blastomere numbers and (ii) considering the proportion of ICM cell populations that expressed octamer-binding transcription factor 4 (OCT4/POU5F1), a pluripotency-associated gene (Khan et al., 2012). In order to identify the changes to gene expression associated with the cellular alterations evidenced after thawing, a transcriptome analysis was performed on fresh isolated ICM derived under different IVP conditions. Finally, in order to confirm gene expression changes relative to cell death and apoptosis, the level of ICM cell death in blastocysts at day 7 post-fertilization was assessed by quantifying terminal deoxynucleotidyl transferase-mediated dUTP nick-end labeling (TUNEL) signals.

## Materials and Methods

### Embryo Production

#### *In vitro* Blastocyst Production

*In vitro* embryos were produced as previously described (Nuttinck et al., 2017). Bovine ovaries were collected at the slaughterhouse and COCs were aspirated from 3 to 6 mm antral follicles. Only oocytes surrounded by more than three compact layers of cumulus cells were selected. The COC were randomly assigned to three IVP treatment groups : serum-containing IVP conditions including the serum supplementation of IVM and IVD culture media (“SC”), experimental serum-free IVP conditions (“SF”), and experimental serum-free IVP conditions associated with the PGE2 supplementation of IVM and IVF culture media (“SF + PGE2”). Following three washes in HEPES-buffered M199 (Sigma), groups of up to 50 immature COCs were transferred to 4-well plates (Nunc, Roskilde, Denmark) containing 500 μl maturation medium at 38.5°C in a water-saturated atmosphere under 5% carbon dioxide. The “SC” maturation medium consisted in TCM199 (Sigma) containing 10% (v/v) fetal calf serum (Invitrogen), supplemented with 10 ng/ml epidermal growth factor (mouse EGF, Sigma), 1 μg/ml 17β-estradiol (Sigma), 10 μg/ml FSH and 10 μg/ml pLH [Stimufol and pLH purified from porcine pituitaries (Reprobiol, Belgium)]. The experimental maturation medium (“SF”) consisted in TCM199 (Sigma) supplemented with 10 ng/ml epidermal growth factor (mouse EGF, Sigma) and 6 mg/ml bovine serum albumin (BSA). After a 22 h culture period, the COCs underwent *in vitro* fertilization (IVF) as previously described (Parrish et al., 1988). The IVM/IVF steps in the experimental treatment groups were performed in the presence (“SF + PGE2”) or absence (“SF”) of 1 μM PGE2 (Cayman Chemicals), as previously described (Nuttinck et al., 2017). A stock solution of 1 mM PGE2 was prepared and stored at –20°C until use. On the day of the experiment, the stock solution was further diluted to 1:1000 in the “SF + PGE2” culture medium to reach the 1 μM concentration. The same volume of DMSO was added to the IVM/IVF culture media for the “SF” treatment group, to reach a final concentration of 0.5%, v/v. All fertilization procedures were performed using the same batches of IVF medium and the same frozen ejaculate from one bull of proven fertility (ALLICE, France). After IVF, presumptive zygotes were denuded and transferred into 50 μl droplets (under paraffin oil) of embryo development medium. The “SC” IVD culture medium consisted in synthetic oviduct fluid (SOF Minitüb 19990/0040), 5% FCS (v/v) (MP Biomedical), 2% BME amino acid solution (v/v) (Sigma), 1% MEM non-essential amino acid solution (v/v) (Sigma), 3 mM sodium pyruvate (Sigma), and 6 mg/ml BSA (Sigma). The experimental serum-free IVD culture medium consisted in modified SOF supplemented with the same reagents except the serum (Holm et al., 1999). The embryos were cultured for seven days at 38.5°C in a water-saturated atmosphere under 5% CO_2_/5% O_2_/90% N_2_. The rate of blastocyst development was determined at the end of culture. The day of fertilization was considered as day 0. At day 2 post-fertilization (2 dpf), non-cleaved (single-cell) oocytes were separated from those that had cleaved. For the cryotolerance experiment (six independent replicates), embryos recovered on day 7 of IVD culture were graded according to their morphological appearance and the Manual of the International Embryo Transfer Society (Robertson and Nelson, 1998). Only grade-I 7 dpf blastocysts (larger blastocoel volume, thinner zona pellucida, many small compacted cells in ICM) were selected and subjected to the conventional slow-freezing procedure. For the ICM transcriptomic experiment (four independent replicates), grade-I 7 dpf blastocysts were subjected to immunosurgery at the end of the IVD period in order to isolate the ICM from the trophectoderm compartment, according to a protocol inspired by Hosseini et al. (2015). The zona pellucida were removed mechanically using homemade microcapillaries and a micro scalpel (Bioniche, Ultra Sharp Splitting blades, ESE020). The zona-free blastocysts were incubated for 1 h at 38.5°C in Dulbecco’s modified eagle medium (DMEM; Gibco, United States) containing rabbit anti-bovine whole serum (Sigma) in a ratio of 1:15. After washing in PBS, the samples were incubated in DMEM containing non-diluted guinea pig complement (Sigma) for 7 min at 38.5°C. After further washing in PBS, the ICM and trophectoderm were dissociated mechanically by drawing through a fine pipette, and then washed in nuclease-free PBS. The isolated ICM were stored in pools of 8–11 units at –80°C until RNA sequencing analysis. For the embryonic apoptosis experiment (two independent experiments), grade-I 7 dpf blastocysts derived under “SC” and “SF + PGE2” culture conditions were harvested and fixed for 1 h at 4°C in 2.5% (w/v) paraformaldehyde (Sigma) in PBS, then transferred to PBS and stored at 4°C until the TUNEL assay.

#### *In vivo* Embryo Production-Animals

Artificial insemination (AI)-derived embryos were included in the experiments on the immunocytochemistry localization of PTGS2/OCT4 expression in order to establish the expression profile of OCT4 by ICM cells between days 8 and 11 of *in vivo* development. The animals were managed in accordance with the European Community Directive 2010/63/EU and under the license granted by the National Research Institute for Agriculture, Food and the Environment (INRA-UCEA). All experiments were carried out at the INRAE experimental farm (registered N°FRTB910 in the national registry for experimental farms). All protocols were approved by the local Animal Care and Use Committee and, for the later collection periods, by the local Ethics Committee (registered as N°12/086 in the National Ethics Committee registry). The embryo donor animals (*n* = 7) were Holstein heifers (4.24 ± 0.78 years), specifically intended for embryo production. All females were housed in the same building and fed the same diet (grass silage, hay, straw, concentrates). They were all cycling before the start of the protocol, and cyclicity was determined by repeated observations of estrus behaviors. In addition, each heifer was equipped with a collar to monitor their activity (Heatime Collars). Estrous synchronization, superovulation, AI and transcervical embryo collection procedures were performed as previously described (Richard et al., 2015). Embryo production sessions were repeated twice in order to obtain a minimum of ten embryos per developmental stage. All AI procedures were performed using frozen semen from the same bull (ALLICE, France). Upon transfer, in vivo embryo collection was carried out from recipients at Day 8, 9, or 11 post-insemination (2, 3, and 2 embryo donors, respectively). At least two cycles were allowed to elapse between two embryo collections. After recovery, the embryo samples were prepared for PTGS2/OCT4 immunocytochemistry analysis.

### Embryo Cryopreservation and the Assessment of Cryotolerance

#### Slow Freezing

Slow freezing was conducted as described elsewhere with some minor modifications (Gupta et al., 2017). Groups of a maximum of 10 grade-I 7 dpf blastocysts per treatment were transferred through ten successive 200 μl washing droplets of embryo holding medium (EHM, IMV Technologies) at room temperature. The embryos were then equilibrated in cryoprotectant freezing solution [1.5 M ethylene glycol (IMV) + 0.1M sucrose (Sigma)] through two successive 200 μl droplets for 5 min each, at room temperature. Within 10 min, each group of a maximum of ten blastocysts was loaded into 0.25 ml straws (IMV Technologies), sealed and kept in the controlled rate freezing unit (model CL-8800, CryoLogic Pty. Ltd., Australia) already cooled at –6°C. Seeding was induced after 5 min. The straws were maintained at –6°C for another 5 min and then cooled at a rate of 0.3°C/min down to –32°C, before being stored in liquid N2 for several weeks before warming.

#### Warming and Post-thaw Blastocyst Culture

The straws were held in the air for 20 s and then immersed in a water bath at 20°C for 2 min. Thawed blastocysts were retrieved, rinsed twice in EHM and counted before a further 3-day culture period in the serum-supplemented or experimental serum-free IVD culture media, depending on the treatment group. Post-cryopreservation survival was defined as a re-expansion of the blastocoele, which was assessed at 24, 48, and 72 h post-thawing, and the hatching rate was assessed at 72 h post-thawing. At the end of culture, surviving blastocysts were prepared for PTGS2/OCT4 immunocytochemistry analysis.

### PTGS2/OCT4 Immunocytochemistry

Day 8, 9, and 11 post-AI embryos and 72 h post-thawed embryos underwent the immunohistochemistry detection of PTGS2 and OCT4 as previously described (Nuttinck et al., 2002; Khan et al., 2012). Briefly, the embryos were permeabilized for 1 h in PBS with 0.5% (v/v) Triton X-100 (Sigma) and washed twice in PBS. These permeabilized embryos were then blocked in 0.01% PBS/triton X-100 (v/v) containing 2% (w/v) BSA for 1 h at 37°C and incubated over night with a mixture of two primary antibodies : mouse monoclonal anti-COX2 (dilution 1:200) (mAB294, gift from Dr. Christophe Creminon, C.E.A., Gif-sur-Yvette, France) and rabbit polyclonal anti-OCT4 (dilution 1:150) (Ab18976, Abcam). After several washes, the embryos were incubated with a mixture of two secondary antibodies: fluorescein isothiocyanate-conjugated donkey anti-mouse IgG antibody (dilution 1:300) (715-O95-151, Jackson ImmunoResearch; Interchim) and cyanine Cy^TM3^-conjugated donkey anti-rabbit IgG antibody (dilution 1:200) (711-165-152, Jackson ImmunoResearch; Interchim). Omission of the primary antibody was used as a negative control. The chromatin was then counterstained with DAPI (diluted 1:1000 in PBS). The embryos were then observed using an inverted ZEISS AxioObserver Z1 microscope equipped with an ApoTome slider, a Colibri light source, and an Axiocam MRm camera controlled by Axiovision software (version 4.8) (MIMA2 imaging platform, INRAE, Jouy-en-Josas, France^1^). ICM cells were identified indirectly from the immunocytochemistry location of expression sites for prostaglandin G/H synthase 2 (PTGS2), a trophectoderm-specific protein seen during preimplantation development in ruminants (Charpigny et al., 1997; Sandra et al., 2017). The total numbers of cells per trophectoderm and per ICM, and the numbers of OCT4-labeled ICM cells, were all estimated using ImageJ software^2^.

### Analysis of ICM Apoptosis Using TUNEL Assay

Grade-I 7 dpf blastocysts derived from “SC” and “SF + PGE2” IVP (*n* = 29 and *n* = 24, respectively) underwent a TUNEL assay as previously described (Nuttinck et al., 2017). The embryos were permeabilized as described above. As positive controls, some embryos were incubated in 50 units DNase/ml PBS (RQ1; Promega) for 30 min at 37°C and then washed twice in PBS before being incubated in 2 μl terminal deoxynucleotidyl transferase, 10 μl fluorescein-conjugated dUTP and 90 μl equilibration buffer (DeadEnd Fluorometric TUNEL System, Promega) for 1 hour in the dark. The embryos were transferred to a 2X standard saline citrate (SSC) buffer to halt the reaction and then washed in PBS. The chromatin was counterstained with DAPI (diluted 1:1000 in PBS) and the embryos were then observed as described above. Using ImageJ software^2^, the area of the ICM was outlined manually on the image stack of the blastocyst, thus determining the region of interest (ROI) for each embryo. The ROI was then used to determine the total integrative density for the TUNEL signal and the ratio of TUNEL positive cells to total ICM cells. Three-dimensional (3D) projections of DAPI/TUNEL labeling were reconstructed using IMARIS 8.3.1 software (Bitplane, Switzerland).

### RNA Sequencing of Isolated ICM

For each IVP treatment group, pools (“SC,” *n* = 3; “SF,” *n* = 4; “SF + PGE2,” *n* = 4) of 8–11 isolated ICM were analyzed. Total RNA was extracted using the Arcturus PicoPure RNA isolation kit (Applied Biosystems, Life Technologies), according to the manufacturer’s instructions. The remaining total RNA samples were kept to enable the performance of RT-qPCR in order to confirm any differentially expressed genes identified by RNA sequencing analysis. RNA sequencing libraries were prepared and sequenced on the GenomEast Platform (IGBMC^3^). These libraries were built using the Clontech SMART-Seq v4 Ultra Low Input RNA kit for Sequencing. Full-length cDNA were generated from 4 ng total RNA using the Clontech SMART-Seq v4 Ultra Low Input RNA kit for Sequencing (Takara Bio Europe, Ozyme) according to the manufacturer’s instructions, with 10 cycles of PCR for cDNA amplification by Seq-Amp polymerase. 600 pg pre-amplified cDNA were then used as the input for Tn5 transposon tagmentation using the Nextera XT DNA Library Preparation Kit (Illumina), followed by 12 cycles of library amplification. After purification with Agencourt AMPure XP beads (Beckman-Coulter, France Genomics), library sizes and concentrations were assessed by capillary electrophoresis. Sequencing was performed on an Illumina HiSeq 4000 with 50 bp paired-end reads. Image analysis and base calling used RTA 2.7.3 and bcl2fastq 2.17.1.14.

### Quantitative RT-PCR

RT-qPCR was carried out to verify the effect of IVP conditions on the expression levels of a few genes related to the apoptosis pathway (MYBBP1A, GADD45A, DDIT3, HERPUD1) and the epigenetic regulation pathway (JARID2, DNMT1). The ACTB housekeeping gene was included in the assay as a reference. Reverse transcription and real-time PCR quantifications were performed as previously described (Nuttinck et al., 2011). First-strand cDNA was synthesized from total RNA using an oligo-dT primer and Super Script II reverse transcriptase (Invitrogen, Life Technologies). Transcript abundance was quantified with an ABI Prism 7000HT (Applied Biosystems) using Takara Ex Taq DNA Polymerase (Takara Bio). For all factors, five log dilutions of the appropriate purified cDNA were included in each assay and used to generate a standard curve. Following PCR, a melting curve was performed on the amplified products to ensure that only specific PCR amplicons had been obtained and quantified. The expression of all target mRNAs was determined in each sample during independent assays. The PCR value was expressed as a ratio to ACTB mRNA for each sample. The primer sequences were designed using the Primer3Plus tool^4^. Primer sequences, product sizes and accession numbers are provided in the Supplementary Table SMM1.

### Data Processing and Statistics

Statistical analysis was performed under R software (v3.6.4). In experiments to evaluate (i) the cleavage rate, (ii) the blastocyst rate, and (iii) the post-thaw survival rate, data were analyzed using a generalized linear mixed-effects model (GLMM, *glmer* function, ‘lme4-package’ version 1.1-21 and ‘lmerTest-package’ version 3.1-2 using a binomial distribution with logit link). The different embryo IVP sessions were considered as random variables. The different IVP conditions (“SC,” “SF” and “SF + PGE2”) and days of post-thaw culture (day-1, day-2, and day-3) were fixed factors. Estimated marginal means (least-squares means) and post-hoc tests were performed using the ‘emmeans’ package, version 1.4.5, with *emmeans* and *pairs* functions.

In the experiments on post-thawing embryo survival, the frequency of ICM cells expressing OCT4, the total number of trophoblastic cells and the quantification of signals from the TUNEL experiments analyzed using a linear mixed-effects model (GLMN, with the *lmer* function ‘lme4-package,’ version 1.1-21). The IVP condition was the fixed factor and the embryo production session the random effect. Contrasts were determined using the ‘emmeans’ package with *emmeans* and *pairs* functions. For the quantification of gene expression by RT-qPCR, the cDNA copy numbers were expressed relative to ACTB cDNA copy numbers. Analysis of variance with the lm function of R Stats Package (version 3.6.1) was used to test the significance of any differences in expression between groups. In the Figures, the results are shown as least-squares means with standard errors. Results were considered statistically significant at a *P*-value of less than 0.05. A *P*-value lower than 0.15 was considered as a tendency towards significance.

For transcriptomic data derived from isolated ICM, the exploratory analysis and differential expression of gene levels were performed using RNAseq workflow, as previously described (Love et al., 2015) and the updated version^5^. The *Salmon* method (Patro et al., 2017) was used to quantify transcript abundance. The cDNA sequence database for *Bos taurus* was obtained from the Ensembl genome browser (release-98101; Bos_taurus.ARS-UCD1.2.cdna.all.fa) and used to build a reference index for the bovine transcriptome (Patro et al., 2017). After quantifying the RNA-seq data, the *tximport* method (Soneson et al., 2015) (R package version 1.8.0) was used to import *Salmon’s* transcript-level quantifications into the downstream *DESeq2* package (R package, version 1.20.0) to analyze differentially expressed genes (DEGs) using the statistical method proposed (Love et al., 2014). BioMart from Ensembl (release 101)^6^ was used to extract gene names and gene descriptions via the Cow genes ARS-UCD1.2 database. To explore the transcriptome of ICM from the three groups, between-class principal component analysis (bcPCA) was performed with ade4 (R package version 1.7-15) using the variance stabilizing transformation output files from DESeq2. Differentially expressed genes were identified by DESeq2 through comparisons between IVP treatment groups (“SC,” “SF,” and “SF + PGE2”) with an adjusted *p*-value of 0.05.

### Gene Ontology

Lists of genes expressed by ICM and contributing to bcPCA dimensions, together with lists of differentially expressed genes under the three types of treatment, were annotated using the PANTHER classification system (Protein Analysis THrough Evolutionary Relationships version 14.0^7^). Pathway enrichments and GO terms were determined using PANTHER overrepresentation tests with all genes from the entire Bos taurus genome as the reference.

## Results

### *In vitro* Blastocyst Development

Six independent sessions of *in vitro* embryo production, each including the three IVP conditions (“SC,” “SF,” “SF + PGE2”), were performed in order to produce Grade-I blastocysts to be evaluated for cryotolerance. *In vitro* development parameters were recorded. As shown in Table 1, cleavage rates were significantly lower in the “SF” and “SF + PGE2” treatment groups. However, the proportion of embryos developing to the blastocyst stage was similar in all treatment groups. While no difference in Grade-I blastocyst rates was evidenced between the “SC” and “SF + PGE2” treatment groups, the “SF” treatment group showed a significantly lower rate when compared with the “SC” group (*P* < 0.05) and tended to be lower than the “SF + PGE2” group (*P* = 0.09).

**TABLE 1:** *In vitro* development of bovine embryos derived from experimental serum-free IVP conditions, supplemented with PGE2 (SF + PGE2), or not (SF) during IVM and IVF, in comparison to the standard serum-containing (SC) IVP system.

		2 dpf*	7 dpf**	
IVP treatment	Cultured oocytes	Cleaved oocytes	Total blastocysts	Grade-I blastocysts
	*N*			
“SC”	334	0.93(0.01)^a^	0.41(0.03)^a^	0.31(0.03)^a^
“SF”	331	0.86(0.02)^b^	0.35(0.03)^a^	0.23(0.03)^b^
“SF + PGE2”	394	0.82(0.02)^b^	0.38(0.03)^a^	0.30(0.03)^ab^

* Proportion of cleaved oocytes relative to the total number of cultured oocytes and ** proportion of blastocysts relative to the total number of cleaved oocytes. dpf, days post-fertilization.

*Values are expressed as LS means (SE), least-squares means (standard error), ^*a,b*^different letters within the same column are statistically different (*p* < 0.05).*

### Cryotolerance

**FIGURE 1:**
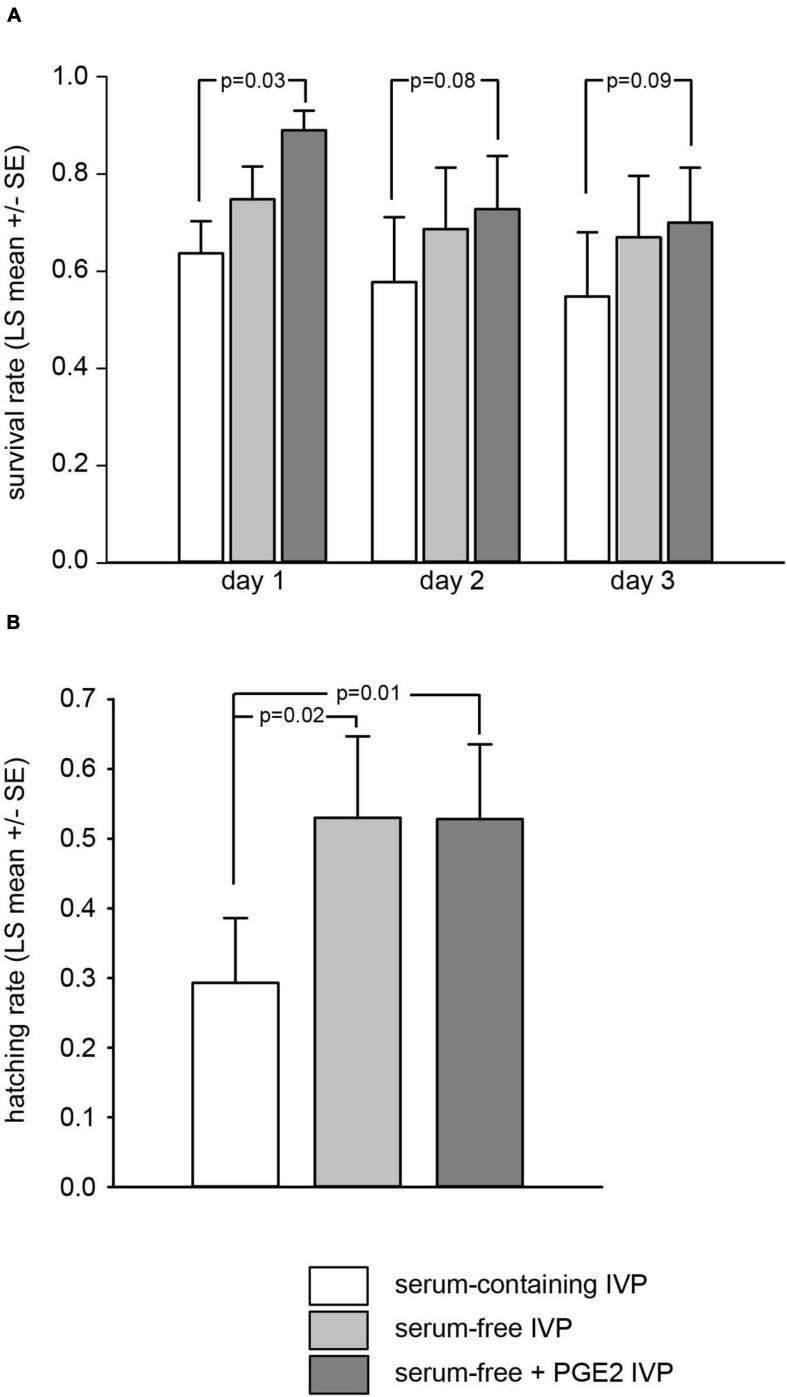
Culture conditions used during the *in vitro* production of bovine blastocysts alter embryo survival on days 1, 2, and 3 **(A)** and hatching rate on day 3 **(B)** after thawing. Proportion of embryos relative to the total number of thawed blastocysts. Values are represented as LS mean ± SE.

A total of 182 thawed grade-I blastocysts (65, 47, and 70 in the “SC,” “SF,” and “SF + PGE2” treatment groups, respectively) were cultured for three days, during which survival parameters were noted. After one day of post-thaw culture, the survival rate of blastocysts derived from the “SF + PGE2” culture condition was significantly higher than in the “SF” or from “SC” treatment groups (*P* = 0.03; Figure 1A). This trend remained throughout the three days post-thaw culture period although no significant differences were found between the treatment groups on days 2 and 3 after thawing. In addition, the day 3 post-thaw hatching rate was affected by IVP conditions. The hatching rate of thawed blastocysts derived from the “SF” and “SF + PGE2” treatment groups was significantly higher than that seen in the “SC” treatment group (*P* = 0.02 and *P* = 0.01, respectively; Figure 1B).

### ICM Size and OCT4 Expression

**FIGURE 2:**
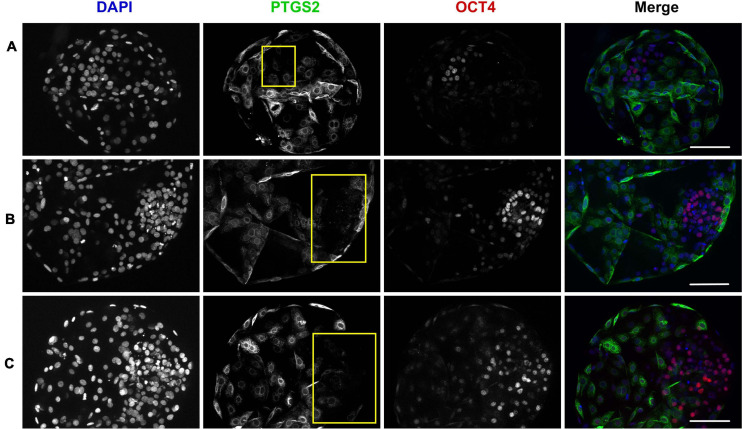
Photomicrographs show representative image stacks of surviving day 3 post-thaw bovine embryos with low **(A)**, medium **(B)**, and high **(C)** proportions of OCT4 positive cells in the ICM. Embryos are stained for PTGS2 (green) and OCT4 (red) expression by immunofluorescence. The chromatin is counterstained with DAPI (blue). The ICM is outlined in yellow. (Scale bars, 100 μm).

**FIGURE 3:**
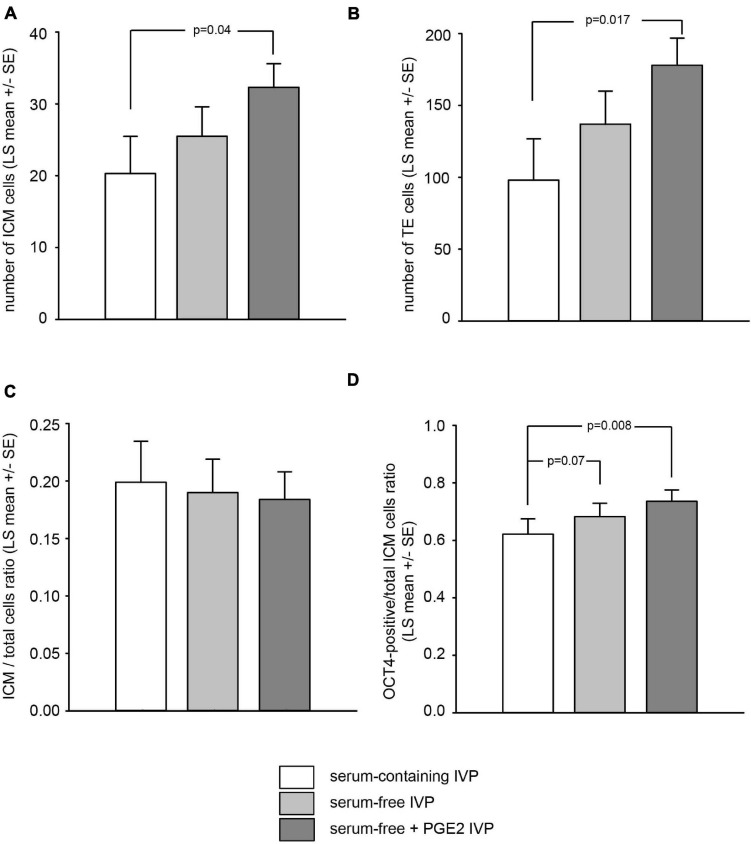
Culture conditions used during the *in vitro* production (IVP) of bovine blastocysts alter the total number of ICM cells **(A)**, trophectoderm cells **(B)**, and the proportion of OCT4-positive ICM cells relative to the total number of ICM cells **(D)** on day 3 after thawing. The proportion of ICM cells relative to the total number of blastomeres was unaffected by IVP conditions **(C)**. Samples sizes for embryos derived from “serum-containing,” “serum-free” and “serum-free + PGE2” *in vitro* production conditions were *n* = 18, 25, and 43, respectively. Values are represented as LS mean ± SE.

At the end of the three days of post-thaw culture, a total of 86 surviving embryos (18, 25, and 43 in the “SC,” “SF,” and “SF + PGE2” treatment groups, respectively) were analyzed for their OCT4/PTGS2 protein expression in order to describe the ICM in terms of total cell numbers and the proportion of OCT4-positive cells. Being strictly localized in the trophectoderm, the immunolabeling of PTGS2 enabled us to identify the ICM cells indirectly. Before this, OCT4 expression was explored in fresh AI-derived embryos at days 8, 9, and 11 of *in vivo* development (*n* = 11, 14, and 12, respectively). As shown in Supplementary Figure SR1, all ICM cells were immunopositive for OCT4 regardless of the *in vivo* developmental stage examined. Initially weak at day 8, OCT4 immunolabeling developed to become intense at day 11. Surviving day 3 post-thaw embryos were then analyzed (Figure 2). While the proportion of embryos containing an ICM was similar in all IVP treatment groups (83, 77, and 90% in the “SC,” “SF,” and “SF + PGE2” treatment groups, respectively), the ICM parameters recorded were affected by the culture conditions. The total numbers of ICM and trophectoderm cells were significantly higher in embryos in the “SF + PGE2” treatment group than in those from the other two IVP treatment groups (*P* = 0.04 and *P* = 0.017, respectively; Figures 3A,B). No difference in the ICM/total blastomeres ratio was evidenced between the treatment groups (Figure 3C). In addition, the proportion of OCT4-positive cells was significantly higher in ICM derived from the “SF + PGE2” treatment group (*P* = 0.008) and tended to be higher in ICM from the “SF” group (*P* = 0.07) than the “SC” group (Figure 3D).

### ICM Transcriptome

Four independent sessions of *in vitro* embryo production, each including the three IVP conditions, were performed in order to produce pools (3, 4, and 4 for the “SC,” “SF,” and “SF + PGE2” treatment groups, respectively) of 8–11 fresh dissected ICM. Their transcriptomic profiles were established by RNA sequencing. The numbers of raw reads (paired-end) ranged from 63 to 94 million per sample.

#### Between-Class PCA Analysis

**FIGURE 4:**
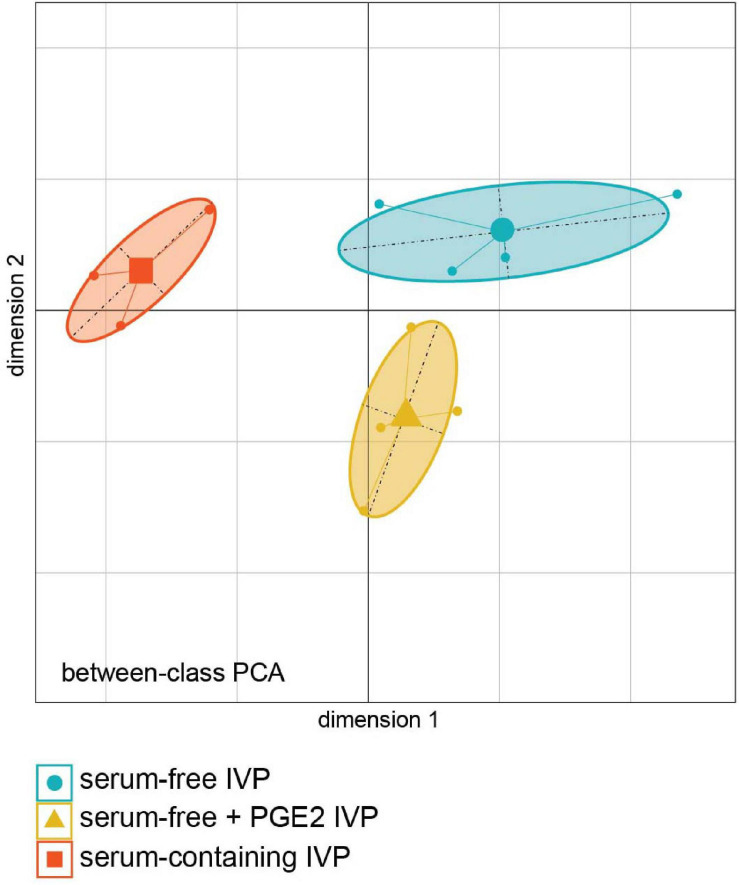
Principal Component Analysis combined with duality diagram functions (dudi) was used to perform Between-Class Analysis with respect to ICM sample assignment to IVP treatment groups. ICM derived from day-7 grade-I blastocysts *in vitro* produced under “serum-containing,” “serum-free,” and “serum-free + PGE2” IVP conditions. Monte Carlo test based on 999 permutations demonstrates that groups are significantly different (*Monte Carlo P-value* = 0.006). (PCA plot was generated using the Ade4 R package).

Transcriptomic analysis showed that the three ICM groups were clearly separated by the between-class PCA (bcPCA, Figure 4). The first dimension separated ICM from the “SC” treatment group (left in Figure 4) from those in the “SF” and “SF + PGE2” treatment groups (right in Figure 4), while these two latter groups were separated according to the second dimension. The genes contributing at least 70% to each of the two components are shown in Supplementary Table SR1. The bcPCA analysis revealed that 36% of total gene expression variability was attributed to the three different treatment groups. The statistical significance of this separation between the treatment groups was validated by the permutation test (*P* = 0.006) (Supplementary Figure SR2) and enabled further investigation of the bcPCA.

The genes contributing to the two dimensions were explored using the PANTHER statistical overrepresentation test (Protein Analysis THrough Evolutionary Relationships version 14.0, see footnote 7) with the Slim-Biological Process classification. Table 2 summarizes the GO terms associated with genes contributing to the first dimension and enabling separation of the “SC” group. Genes encoding proteins for cell death (GO:0008219) and the apoptotic process (GO:0006915) were over-represented in ICM isolated from embryos produced under these IVP conditions. Two other groups of genes coding for unsaturated fatty acid biosynthetic process proteins (GO:0006636) and the membrane lipid biosynthetic process (GO:0046467) were enriched in the ICM of embryos from “SC” cultures. Finally, genes involved in phagocytosis (GO:0006909), the carbohydrate metabolic process (GO:0005975) and organic anion transport (GO:0015711) were enriched. Table 3 shows the GO terms enrichment rates obtained with genes positively correlated with the first dimension and associated with ICM from embryos derived under “SF” and “SF + PGE2” IVP conditions. A set of genes coding for proteins related to the cell cycle process and cell cycle DNA replication was over-represented [DNA replication initiation (GO:0006270); double-strand break repair via break-induced replication (GO:0000727)]. A second set of genes was related to ribosomal small subunit biogenesis (GO:0042274). These genes are involved in the rRNA processing (R-BTA-72312) and protein translation (R-BTA-72766) pathways. Genes associated with Gap junction trafficking and regulation (R-BTA-157858), were also over-represented in the ICM of embryos from the two groups of experimental serum-free IVP, with and without PGE2. The second dimension separated the “SF + PGE2” group from the two other groups. The over-represented genes associated with the “SF + PGE2” IVP treatment group were assigned to three main GO terms (Table 4): intermediate filament cytoskeleton organization (GO:0045104); leukocyte cell–cell adhesion (GO:0007159) which forms a part of cell adhesion (GO:0007155), and transporter activity (GO:0005215). It was quite difficult to identify GO terms that reflected unambiguous enrichment with the 90 genes positively correlated to dimension 2 and associated with the ICM from the “SC” and “SF” groups (Table 5). In any case, some genes in the Rho GTPase cycle contributed to enriching these signaling pathways (BTA-194840).

**TABLE 2:** Enrichment of genes contributing to the first dimension of between-class PCA (negatively correlated) and associated with ICM from the “SC” IVP treatment group.

PANTHER GO-slim biological process	Fold enrichment	FDR	Genes
Cell death (GO:0008219)	3.51	1.51E-03	DDIT3, EXOG, LGALS3, ATM, TIGAR, LATS1, AIFM2, ANXA1, PHLDA3, PPIF, BDNF, RETREG1, DNASE2, CCN2, CCN3, HERPUD1
Positive regulation of cell death (GO:0010942)	5.24	1.70E-02	
Regulation of cell death (GO:0010941)	5.45	3.16E-03	
Programmed cell death (GO:0012501)	3.4	2.85E-03	
Positive regulation of programmed cell death (GO:0043068)			
Apoptotic process (GO:0006915)	3.12	9.59E-03	
Positive regulation of apoptotic process (GO:0043065)	5.45	1.52E-02	
Unsaturated fatty acid biosynthetic process (GO:0006636)	17.36	1.58E-02	PTGES, MGST1, ANXA1
Membrane lipid biosynthetic process (GO:0046467)	4.79	2.85E-02	PIGN, ST6GALNAC4, NAGA, PIGM, PGAP1, NEU1, GLA, DEGS1, SGMS2, UGCG, SAMD8, PIGM
Sphingolipid metabolic process (GO:0006665)	6.17	9.90E-03	
Ceramide metabolic process (GO:0006672)	9.26	2.17E-03	
Phagocytosis (GO:0006909)	4.55	3.47E-02	BIN2, MET, MERTK, ANXA3, ENSBTAG00000001219, ANXA1, XKR8
Carbohydrate metabolic process (GO:0005975)	3.86	2.96E-03	PIGN,ST6GALNAC4, NAGA, PIGM, PGAP1, NEU1, GLA, UGCG, PIGM
Glycolipid metabolic process (GO:0006664)	7.62	1.69E-03	
Organic anion transport (GO:0015711)	3.5	3.04E-02	SLC16A7, ENSBTAG00000049434, CTNS, SLC38A9, SLC38A6, SLC36A2, SLC17A5, CLN3, SLC10A1, ANXA1
Carboxylic acid transport (GO:0046942)	3.51	1.51E-03	

**TABLE 3:** Enrichment of genes contributing to the first dimension of between-class PCA (positively correlated) and associated with ICM from the “SF” and “SF + PGE2” IVP treatment groups.

PANTHER GO-slim biological process	Fold enrichment	FDR	Genes
Double-strand break repair via break-induced replication (GO:0000727)	18.05	5.85E-02	MCM3, GINS2, MCM2, MCM5, MCM7
Ribosomal small subunit biogenesis (GO:0042274)	5.55	3.02E-02	RPS15, TBL3, RPS19, RPS10, RPS16, SURF6, DHX37, ENSBTAG00000007105, HEATR1, RPS21

**PANTHER reactome pathways**	**Fold enrichment**	**FDR**	**Genes**

Gap junction trafficking and regulation (R-BTA-157858)	7.74	4.79E-02	GJA1, TUBA8, TUBA1A, TUBB4A, SRC
Microtubule-dependent trafficking of connections from Golgi to the plasma membrane (R-BTA-190840)	12.38	4.27E-02	
Signaling by EGFR (R-BTA-177929)	7.74	4.98E-02	SH3GL1, SH3GL, PLCG1, UBA52, SRC
Translation (R-BTA-72766)	3.68	1.09E-02	RPLP1, RPL18A, MRPS26, RPS19, RPS10, RPS16, RPL28, UBA52, RPLP2, RPL32, RPL26, RPS23, RPS21
GTP hydrolysis and joining of the 60S ribosomal subunit (R-BTA-72706)	7.12	6.98E-04	
rRNA processing (R-BTA-72312)	4.61	3.42E-04	RPLP1, RPL18A, RPS19, RPS10, RPS16, DHX37, RPL28, NOL6, UBA52, RPLP2, RPL32, RPL26, RPS23, RPS21
Major pathway of rRNA processing in the nucleolus and cytosol (R-BTA-6791226)	4.61	3.73E-04	

**TABLE 4:** Enrichment of genes contributing to the second dimension of between-class PCA (negatively correlated) and associated with ICM from the “SF + PGE2” IVP treatment group.

PANTHER GO-biological process complete	Fold enrichment	FDR	Genes
Intermediate filament cytoskeleton organization (GO:0045104)	12.89	6.49E-03	PLEC, PRPH, MTM1, KRT20, ATP8A2, PKP1, DSP
Cell adhesion (GO:0007155)	3.06	6.23E-03	SPP1, CDH11, SEMA4D, TNFAIP6, LAMA1, COL15A1, GPC4, JAM2, MFGE8, PODXL2, PTPRF, CD24, ADGRG1, PKP1, SULF1, L1CAM, ITGB7, AJUBA, BCAM, NT5E, RAC2, CLSTN3, CLSTN2, DSP, CD24
Leukocyte cell–cell adhesion (GO:0007159)	11.56	2.99E-02	SEMA4D, JAM2, PODXL2, ITGB7, NT5E, RAC2

**PANTHER GO-slim molecular function**	**Fold enrichment**	**Bonferroni**	**Genes**

Transporter activity (GO:0005215)	2.52	1.05E-02	ABCC10, ATP2C2, ATP8A2, ATP8B4, ENSBTAG00000005997, ENSBTAG00000046447, GABRA5, ITPR3, OSBPL6, RANBP17, SLC26A2, SLC26A2, SLC27A1, SLC27A2, SLC28A1, SLC38A2, SLC39A10, SLC39A2, SLC39A4, SLC9A6, SLCO2A1, SLCO4C1, TCIRG1, TMC7, TMEM63A, VDAC3

**TABLE 5:** Enrichment of genes contributing to the second dimension of between-class PCA (positively correlated) and associated with ICM from the “SC” and “SF” IVP treatment groups.

PANTHER GO-reactome pathways	Fold enrichment	FDR	Genes
ER-Phagosome pathway (R-BTA-1236974)	48.38	1.99E-02	BOLA, C1R, JSP.1, MAVS, VAV2
Antigen Presentation: Folding, assembly and peptide loading of class I MHC (R-BTA-983170)	36.86	3.28E-02	
Rho GTPase cycle (BTA-194840)	11.56	4.30E-02	VAV2, A2M, TIAM2, ARHGEF3, ARHGAP15

#### DEGs From DESeq 2

Two-by-two comparisons summarized the numbers of differentially-expressed genes (DEG) between ICM derived from the three groups of embryos (*P*-value < 0.05 and *P*-adjusted < 0.05) (Supplementary Tables SR2, SR3, and SR4, and lists of DEGs in Supplementary Table SR5).

##### DEGs in ICM under “SC” versus “SF” IVP conditions

More than half of the genes overexpressed in ICM from the “SC” treatment group were indicative of a significant enrichment of metabolic processes (catabolic process, GO:0009056; carbohydrate derivative metabolic process, GO:1901135; organonitrogen compound metabolic process, GO:1901564) (Table 6). On the other hand, major enrichment of the genes coding for proteins involved in nucleic acid binding (PC00171) was found in the group overexpressed in ICM from the “SF” treatment group (Table 7). These genes are associated with the cell cycle (GO:0007049), cell cycle DNA replication (GO:0044786), DNA replication (GO:0006260) and recombinational repair (GO:0000724). Among the overexpressed genes evidenced in the “SF” treatment group, an additional set of enriched genes was associated with rRNA processing (GO:0006364) and ribonucleoprotein complex biogenesis (GO:0022613).

**TABLE 6:** Enrichment of genes overexpressed in ICM under “SC” versus “SF” IVP conditions.

PANTHER GO-slim biological process	Fold enrichment	FDR	Genes
Protein localization to cell surface (GO:0034394)	46.5	4.51E-03	PIGK; RIC3; LEPROT
Carbohydrate derivative metabolic process (GO:1901135)	3.07	4.33E-02	GUCY2C; PIGK; NAGA; GNPDA1; DPAGT1; FUCA2; DPAGT1; NOS3; SGSH; UGCG; PANK3; ITM2B; NAGA; C1GALT1; FUCA1; ALDOC
Organic substance catabolic process (GO:1901575)	2.78	6.58E-03	SCP2; FBXL17; CTSL; ENPP1; NAGA; FBXL3; RNF13; PSME4; GNPDA1; LAMP2; PSME4; RIDA; FUCA2; PLA2G4A; CTSZ; NOS3; SGSH; CTSL; NAGA; CTSV; DNASE2; FUCA1; HERPUD1; ALDOC; ENPP3
Catabolic process (GO:0009056)	2.69	3.01E-03	SCP2; FBXL17; CTSL; ENPP1; NAGA; FBXL3; ATG5; RNF13; PSME4; TP53INP1; GNPDA1; LAMP2; GABARAP; PSME4; RIDA; RRAGC; FUCA2; PLA2G4A; CTSZ; NOS3; SGSH; CTSL; NCEH1; NAGA; CTSV; DNASE2; FUCA1; HERPUD1; ALDOC; ENPP3
Cellular catabolic process (GO:0044248)	2.45	3.69E-02	SCP2; FBXL17; CTSL; ENPP1; NAGA; FBXL3; ATG5; RNF13; PSME4; TP53INP1; GNPDA1; LAMP2; GABARAP; PSME4; RRAGC; PLA2G4A; CTSZ; NOS3; SGSH; CTSL; CTSV; DNASE2; HERPUD1; ENPP3
Organonitrogen compound metabolic process (GO:1901564)	1.71	4.72E-02	PRCP; GUCY2C; SEC11C; FBXL17; PIGK; PKIA; SDCBP; CTSL; STK38L; NAGA; FBXL3; ATG5; FURIN; RNF13; PSME4; TP53INP1; CPE; LAMP2; GADD45A; GABARAP; DPAGT1; GCH1; PSME4; RIDA; PTPRK; BMPR1B; DPAGT1; CTSZ; DEGS1; NOS3; IFNT; MAP4K3; SGSH; CTSL; PRCP; AKTIP; UGCG; CPXM2; PANK3; ITM2B; CTSV; CCNG1; ARV1; C1GALT1; GSTA3; GCLM; PGGT1B; TSPAN33; RPS6KB1; PRRC1; HERPUD1; ALDOC; RPS6KB1; PARP3

**TABLE 7:** Enrichment of genes overexpressed in ICM under “SF” versus “SC” IVP conditions.

PANTHER GO-slim biological process	Fold enrichment	FDR	Genes
Pre-replicative complex assembly involved in nuclear cell cycle DNA replication (GO:0006267)	44.93	2.61E-05	MCM3; MCM4; MCM2; MCM6; MCM5; MCM7
Double-strand break repair via break-induced replication (GO:0000727)	34.94	8.90E-06	MCM3; CDC45; MCM4; ZSWIM7; MCM2; CDCA5; MCM6; MCM5; MCM7
Mitotic DNA replication (GO:1902969)	33.28	7.09E-04	MCM3; CDC45; MCM4; MCM2; MCM6
Cell cycle DNA replication (GO:0044786)	28.19	8.58E-06	MCM3; CDC45; MCM4; MCM2; DONSON; MCM6; MCM5; MCM7
Nuclear DNA replication (GO:0033260)	28.19	4.29E-06	MCM3; CDC45; MCM4; MCM2; DONSON; MCM6; MCM5; MCM7
DNA replication initiation (GO:0006270)	19.97	8.15E-04	MCM3; CDC45; MCM4; MCM2; MCM5; MCM7
DNA-dependent DNA replication (GO:0006261)	8.29	1.00E-03	MCM3; CDC45; MCM4; TIMELESS; MCM2; DONSON; MCM6; MCM5; MCM7
Double-strand break repair via homologous recombination (GO:0000724)	8.26	2.82E-03	MCM3; CDC45; MCM4; ZSWIM7; MCM2; CDCA5; MCM6; MCM5; MCM7
DNA replication (GO:0006260)	7.09	2.79E-03	MCM3; CDC45; MCM4; TIMELESS; MCM2; DONSON; MCM6; MCM5; MCM7
rRNA processing (GO:0006364)	4.45	1.06E-02	IMP4; RRP1; GAR1; STYXL1; WDR3; SF3A2; RRP1B; NOL10; WDR77; SURF6; DHX37; NOM1; NOL6; PES1; KRI1
Ribosome biogenesis (GO:0042254)	3.22	3.98E-02	IMP4; RRP1; GAR1; STYXL1; WDR3; SF3A2; RRP1B; NOL10; WDR77; SURF6; DHX37; NOM1; NOL6; PES1; KRI1
Cell cycle (GO:0007049)	2.45	4.44E-02	MYB; MCM3; CDC45; MCM4; TUBA8; RAB11FIP3; MST1; E2F4; CDC25A; TIMELESS; MCM2; CCND3; CDCA5; DONSON; MCM6; NDE1; MCM5; MYBL2; MCM7
**PANTHER protein class**			
Nucleic acid binding protein (PC00171)	2	3.17E-02	IMP4; PPP1R8; PCF11; BICC1; GAR1; SF3A2; RAVER1; MCM3; CDC45; MCM4; TRMT2A; RBM14; GTPBP3; E2F4; PIF1; CHTOP; ILF3; PPP1R8; SRSF4; WDR77; SURF6; DHX37; NOM1; MCM2; NOL6; FUS; PES1; MCM6; FUS; MCM5; CHTOP; GTF3C5; MCM7; LIG3

##### DEGs in ICM under “SC” versus “SF + PGE2” IVP conditions

The biological processes most enriched in the genes overexpressed in the “SF + PGE2” group were the histone demethylation process (GO:0016577) and the regulation of histone H3-K9 methylation (GO: 0051570) (Table 8). The DNA replication process (GO: 0006260) was also enriched in the “SF + PGE2” group. Two other significant processes were enriched in the “SF + PGE2” group. The first contributed to enriching the mitotic cell cycle process (GO:1903047), and the second reflected an enrichment of the apoptotic process (GO:0006915).

**TABLE 8:** Enrichment of genes overexpressed in ICM under “SF + PGE2” versus “SC” IVP conditions.

GO biological process complete	Fold enrichment	FDR	Genes
Regulation of histone H3-K9 methylation (GO:0051570)	22.76	3.21E-02	MYB, JARID2, DNMT1, KDM4A
Histone demethylation (GO:0016577)	22.13	9.12E-03	RIOX1, KDM8, JARID2, KDM4C, KDM4A
DNA replication (GO:0006260)	8.07	4.10E-03	POLD1, DHX9, PIF1, CHAF1A, TIMELESS, MCM2, DONSON, MCM6, POLD1, CHTF8
Mitotic cell cycle process (GO:1903047)	3.81	3.12E-02	MYB, FBXL18, KDM8, TUBA8, NUP6,2 MCM2, CTDP1, DONSON, MCM6, INCENP, ZFYVE19, RCC1, CHTF8, KAT14, TACC3
Apoptotic process (GO:0006915)	3.46	1.60E-02	MYBBP1A, XAF1, FAIM, CAV1, EPHA2, GSN, XAF1, CIDEB, ELMO2, MCM2, SLC25A6, CYP1B1, CHIA, CDCA7, GLI3, BCL2L14
DNA metabolic process (GO:0006259)	3.23	3.09E-02	POLD1, PIF1, CHAF1A, RAD54L, TIMELESS, ZSWIM7, TRIM28, DNMT1, MCM2, CHRNA4, DONSON, MCM6, DNMT1, ZBTB48, POLD1, MTA2
Cell cycle (GO:0007049)	3.22	9.26E-03	MYB, FBXL18, KLHDC3, KDM8, TUBA8, HAUS5, NUP62, PHGDH, ODF2, CHAF1A, RNF212B, RAD54L, TIMELESS, MCM2, CTDP1, DONSON, MCM6, STRADA, INCENP, ZFYVE19, RCC1 CHTF8 KAT14 TACC3

Compared to the “SF + PGE2” group, only one molecular function category was found to be enriched with the genes overexpressed in the ICM of the “SC” group (Table 9). Thirty-five of the overexpressed genes revealed the enrichment of catalytic activity.

**TABLE 9:** Enrichment of genes overexpressed in ICM under “SC” versus “SF + PGE2” IVP conditions.

PANTHER GO-slim molecular function	Fold enrichment	FDR	Genes
Catalytic activity (GO:0003824)	2.14	3.95E-03	PHKA1, PRCP, SCP2, RPE, ATG3, ABCC5, CPE, GNPDA1, DPAGT1, POLR2K, TIMP2, EIF3H, CDK7, DERA, EXO5, SAR1B, ACER3, PCMT1, DPAGT1, CDC42, CPA4, NAA20, FBXO11, PRCP, PANK3, GTF2H1, PPT1, CTSV, CCNG1, SELENOF, UBE2Q2, ATP6V0B, PGGT1B, FUCA1, ATP6V1A

##### DEGs in ICM under “SF” versus “SF + PGE2” ICM conditions

Only eight genes are differentially expressed between the “SF” and “SF + PGE2” groups when the adjusted *p*-value was considered (*P* < 0.05). Five were overexpressed in the “SF + PGE2” group (H4C7, ADAM22, GUCY2C, ANKRD13B, SYNJ2) and three in the “SF” group (CPA4, GPR50, RGS3).

When account was taken of the unadjusted probability (*P* < 0.05), 382 and 470 genes were overexpressed in “SF” and “SF + PGE2” groups, respectively (Supplementary Table SR5). Genes overexpressed in the “SF + PGE2” group displayed enrichment in members of the C2 domain (BAIAP3 C2CD5, CC2D2A, CPNE8, DOCK1, DOCK4, DOCK5, PLA2G4A, PLCH1, SYTL4, UNC13B, WWC1, WWC3) (IPR035892), which is thought to be involved in calcium-dependent phospholipid binding.

### RT-qPCR Confirmation of Differences in ICM Gene Expression Between IVP Treatment Groups

**FIGURE 5:**
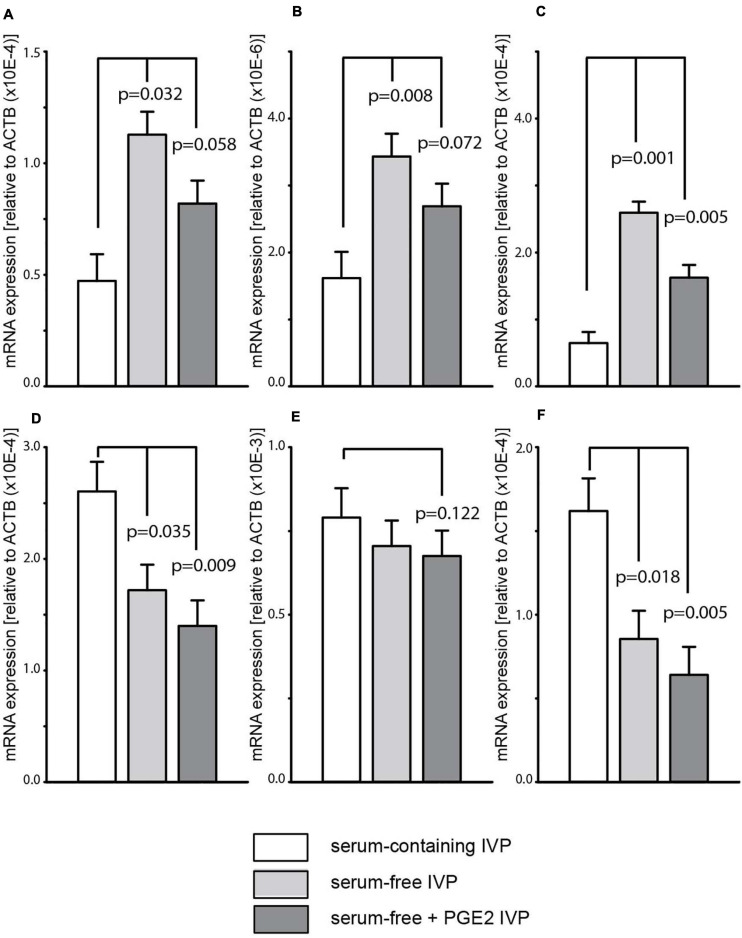
Differences in gene expression between inner cell masses derived from “serum-containing,” “serum-free,” and “serum-free + PGE2” *in vitro* production (IVP) as determined by quantitative PCR. Two genes are related to the epigenetic regulation pathway: JARID2, Jumonji and AT-Rich Interaction Domain Containing 2 **(A)**; DNMT1, DNA (cytosine-5)-methyltransferase 1 **(B)**. Four genes are related to the apoptosis pathway: MYBBP1A, Myb binding protein 1A **(C)**; DDIT3, DNA damage-inducible transcript 3 **(D)**; HERPUD1, homocysteine-inducible, ER stress-inducible, ubiquitin-like domain member 1 **(E)**; GADD45A, Growth arrest and DNA damage-inducible alpha **(F)**. Values are represented as LS mean ± SE of each transcript corrected with the housekeeping gene ACTB.

To verify the RNAseq analysis results, RT-qPCR was carried out to examine the expression levels in ICM derived from the “SC,” “SF,” and “SF + PGE2” groups of two genes related to the epigenetic regulation pathway (JARID2, DNMT1) (Figures 5A,B) and four genes related to the apoptosis pathway (MYBBP1A, DDIT3, HERPUD1, GADD45A = DDIT1) (Figures 5C–F). Overall, the profiles were consistent with the RNAseq findings when ICM expression levels in the “SF” or “SF + PGE2” group were compared to the “SC” group. The expression levels of DNMT1 and HERPUD1 were partially inconsistent with RNAseq analysis results. However, although DNMT1 expression was significantly higher in the “SF + PGE2” group and tended to be higher in the “SF” group in the RNAseq data, RT-qPCR showed the reverse, i.e., significantly higher in the “SF” group” and a tendency to be higher in the “SF + PGE2” group (Figure 5B). While HERPUD1 expression in the RNAseq data was significantly lower in the “SF” group than the “SC” group, RT-qPCR showed no difference between these groups (Figure 5E). The tendency of HERPUD1 expression in RNAseq data to be lower in “SF + PGE2” group than the “SC” group was also found in RT-qPCR data (*P* = 0.122) (Figure 5E).

### TUNEL Quantification

**FIGURE 6:**
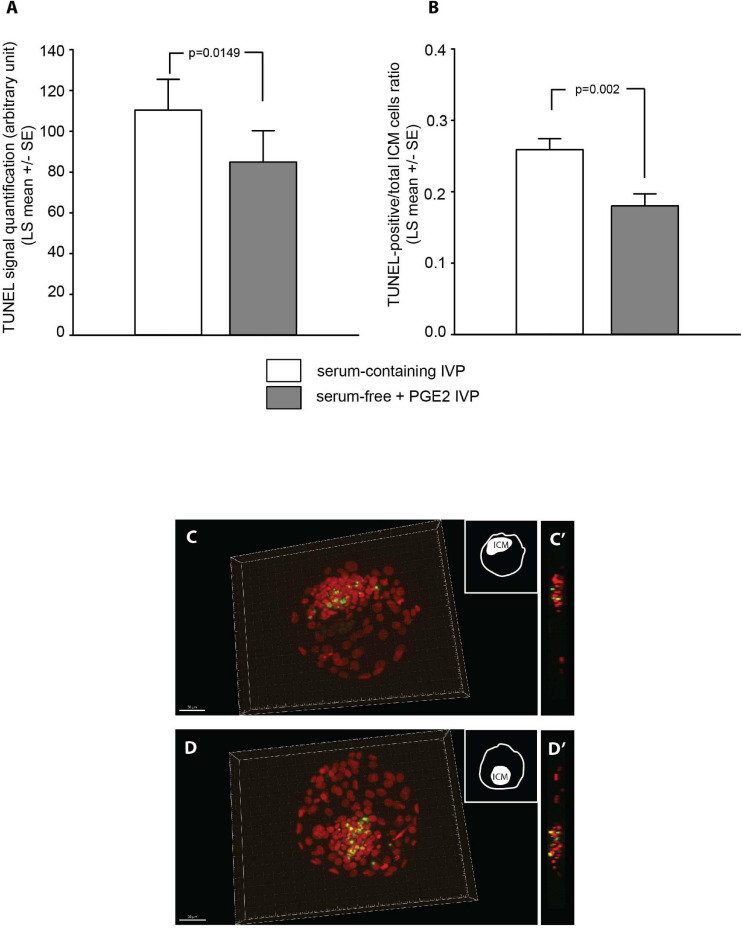
Quantification of TUNEL signals detected in the inner cell mass (ICM) of grade-I 7 dpf bovine blastocysts derived from “serum-containing” and “serum-free + PGE2” *in vitro* production (IVP) **(A,B)**. Samples sizes were *n* = 29 and 24, respectively. Values are represented as LS mean ± SE. Photomicrographs show representative 3D images of low **(C)** and high **(D)** levels of TUNEL signals (green) observed in blastocysts. Photomicrographs **(C’,D’)** represent an orthogonal view of the ICM. The chromatin is counterstained with DAPI (presented in red to contrast with the TUNEL).

The *in situ* detection of DNA fragmentation generated during the apoptotic process was performed using the TUNEL reaction. All 53 Grade-I 7 dpf blastocysts analyzed with fluorescence microscopy exhibited TUNEL. As shown in Figures 6A–D, different levels of TUNEL signal were quantified in ICM according to the IVP conditions (Supplementary Table SR6, ICM cell counts). The signals were significantly lower in blastocysts derived from “SF + PGE2” IVP than in the “SC” group (Figures 6A,B).

## Discussion

Despite its well-known negative impact on the cryotolerance of bovine embryos, serum is still included in the media used in IVP systems because it enables higher developmental rates up to the blastocyst stage. The aim of our study was to compare the developmental and thawing survival performances of bovine blastocysts under experimental serum-free IVP conditions with those produced under the serum-supplemented system. The experimental conditions consisted in removing serum throughout the IVP procedure, combined or not with the PGE2 supplementation of a simple and defined oocyte culture medium. We had previously demonstrated that the PGE2 enrichment of oocyte culture media stimulated cell cycle kinetics and ICM cell survival during subsequent development. We have now shown that embryos produced under experimental PGE-supplemented serum-free conditions during oocyte culture sustained a developmental ability comparable to that of embryos derived from serum-containing IVP. In addition, these embryos displayed improved viability associated with a larger population of ICM cells and higher proportions of ICM cells expressing the OCT4 protein after thawing. Transcriptomic analysis of ICM isolated from morphologically high-grade blastocysts derived under the different IVP conditions highlighted the biological functions associated with the cellular changes noted after thawing.

During the present study, we observed that *in vitro* developmental parameters were affected by the experimental IVP conditions. Lower cleavage rates were recorded when IVP were performed under serum-free conditions, supplemented or not with PGE2. Our results are consistent with those reported by Korhonen et al. (2010). Although the maturation medium they used remained complex and enriched with hormones, they also noted a fall in embryo cleavage rates when just the serum was omitted from IVP culture media. As we had previously reported, the PGE2 supplementation of oocyte culture media did not modify the cleavage rate (Nuttinck et al., 2017). The developmental capacity of cleaved oocytes to form blastocysts appeared to be similar in the different treatment groups, but their ability to reach “good” or “excellent” grade blastocysts differed as a function of culture conditions. The proportion of cleaved oocytes that developed to become morphologically high grade blastocysts remained lower when the experimental serum-free IVP without PGE2 supplementation was used. Interestingly, this proportion increased to become similar to that observed with serum-containing IVP when PGE2 was added to the oocyte culture medium. Previous studies in humans had established that the level of cumulus prostaglandin G/H synthase-2 (PTGS2) expression could be used to predict the quality of subsequent embryo development (McKenzie et al., 2004; Scarica et al., 2019). PTGS2, the inducible isoform of PTGS, catalyzes the first step of the prostaglandin synthesis pathway in follicular somatic cells. LH-induced PTGS2 expression is associated with an enrichment of the oocyte microenvironment with prostaglandins, and particularly PGE2 (Duffy, 2015). A higher level of PTGS2 expression in cumulus cells after *in vivo* maturation correlated to a higher proportion of *in vitro* embryos with an elevated morphological grade and better *in vitro* blastocyst development. Taken together, these previous and present findings reinforce the concept that the degree of periconceptional PGE2 oocyte impregnation participates in defining the trajectory of embryonic development.

For the first time, we focused our investigation of an embryo’s response to different *in vitro* environments on changes to the transcriptomic profile of the ICM, i.e. that part of the blastocyst giving rise to the epiblast and then to all future intraembryonic tissues. Both the exploratory data and differential expression analysis enabled us to identify several biological processes occurring in the ICM whose levels of expression were influenced by IVP conditions. The biological processes we highlighted were determined by taking account of the cellular changes revealed during post-thawing embryo cultures. Our data showed that the expression of genes involved in several metabolic processes, including unsaturated fatty acid biosynthesis, membrane lipid biosynthesis, carbohydrate and organonitrogen metabolism, was markedly impacted when using the serum-containing IVP system. Our ICM transcriptome data reinforced previous reports of whole blastocyst transcriptome analyses that compared embryos produced *in vivo* versus *in vitro* under standard serum-containing conditions (Cagnone and Sirard, 2016). Changes to gene expression related to lipid and carbohydrate metabolism were shown to be induced by the IVP procedure. Furthermore, we found that numerous genes related to catabolic activities were overexpressed in ICM derived under serum-supplemented conditions. The assumed increase in ICM catalytic activities induced by serum-supplemented conditions could partly account for the poorer embryo cryotolerance that was evidenced during the post-thawing period. Indeed, lower post-thawing survival and hatching rates were recorded when serum-containing IVP was applied. Overall, the previous and present data emphasize the impacts of both the IVP procedure itself and the composition of the culture medium on the adaptive metabolic response of the embryo to its surrogate environment. The weaker post-thawing performance associated with the serum-containing IVP system may reflect an inappropriate response of the embryo to the stress induced by these culture conditions.

Our gene ontology investigations highlighted changes to gene expression related to cell death and apoptotic processes in ICM when using serum-containing IVP. The co-enrichment of HERPUD1 (homocysteine-inducible, ER stress-inducible, ubiquitin-like domain member 1) and DDIT3 (DNA damage inducible transcript 3) gene expression deserves further study. The concomitant upregulated expression of these two genes had previously been described in mouse embryos destined to die because of an inability to alleviate endoplasmic reticulum stress (Hao et al., 2009). Several overexpressed genes related to the apoptosis pathway were also detected in ICM that derived from PGE2-supplemented serum-free IVP when compared with the transcriptome of ICM produced under serum-containing conditions. These included the MYBBP1A gene that encodes Myb-Binding Protein 1A, a nucleolar transcriptional regulator of particular interest. MYBBP1A is known to play an important role during early development (Mori et al., 2012); its deletion in the mouse leads to embryonic death before blastocyst formation. In addition, the down-regulation of MYBBP1A induces apoptosis and mitotic anomalies in mouse embryonic stem cells. Our transcriptomic data were supported by the results of TUNEL signal quantification which indicated weaker apoptotic activities in ICM derived from PGE2-supplemented serum-free IVP than when using serum-containing IVP. Overall, our findings have demonstrated that IVP culture conditions can modulate the expression of genes potentially involved in embryonic apoptosis. The incidence of embryonic cell death has been shown to strongly impact post-hatching development. By compromising the survival of ICM cells, an imbalance in the expression of pro- and anti-apoptotic genes could affect subsequent preimplantation development.

Furthermore, numerous genes related to DNA replication, DNA metabolism, DNA double-strand break repair and cell cycle processes were found to be overexpressed in ICM derived from experimental serum-free IVP, supplemented or not with PGE2, when compared to serum-containing conditions. Among these, all members of the minichromosome maintenance (MCM2-7) family were found to be overexpressed. The double hexamer assembly of the heterohexameric MCM2-7 helicase complex at replication origins during G1 phase represents a central step in the DNA replication process in eukaryotes (Li et al., 2015). Expression of the MCM family had previously been described during oogenesis and early development in mice. The involvement of this family in chromatin licensing during the initial embryonic cell cycles is widely accepted (Swiech et al., 2007). The increase in replication activity suggested by our ICM transcriptome analysis was supported by our counts of the total number of ICM cells at the end of post-thawing cultures. Indeed, the use of experimental serum-free culture conditions, and particularly those supplemented with PGE2, was associated with larger ICM cell populations in thawed blastocysts. Our new findings have refined our previous data which showed that the amount of PGE2 in the periconceptional microenvironment of oocytes is involved in controlling the embryonic cell population during early development by regulating both the mitotic cell cycle and apoptotic activities (Nuttinck et al., 2011, 2017).

Our transcriptional data analysis has highlighted the overexpression of genes related to the “regulation of histone H3-K9 methylation” and “histone demethylation” processes specifically in ICM that were obtained under PGE2-supplemented serum-free conditions when compared to ICM derived using serum-containing IVP. Epigenetic regulation participates in the complex orchestration of gene expression associated with pluripotency and initial cell lineage specifications during early development. Among the overexpressed genes evidenced, DNMT1 (which codes for the main methyltransferase involved in maintaining methylation imprints) was of particular interest. Genomic imprinting patterns determine the embryonic phenotype. Any deregulation affecting the maintenance of imprinting can result in embryonic developmental defects (Jones and Liang, 2009). DNMT1 was recently shown to contribute also to the repression of retroelements in preimplantation embryos (Min et al., 2020). The timely activation and repression of retroelement expression may be of great significance to early developmental programs and embryo survival. JARID2, another gene that was overexpressed in ICM derived from PGE2-supplemented serum-free IVP, codes for a member of the jumonji demethylase protein family. JARID2 is described as an important component of the Polycom repressive complex 2 (PRC2) which represses a number of key developmental regulators in order to safeguard pluripotency (Lee et al., 2006); it is also thought to be involved in regulating OCT4 and SOX2 expression in bovine embryos (Fu et al., 2017). The overexpressed gene EPHA2 (Ephrin receptor A2) was also of notable interest. EPHA2 belongs to the Ephrin receptor subfamily of the protein-tyrosine kinase family, and in human and mouse embryonic stem cell models it was recently shown to play a functional role in maintaining pluripotency gene expression and restricting the induction of differentiation markers (Fernandez-Alonso et al., 2020). Interestingly, our investigations of post-thawing blastocyst quality showed that the proportion of ICM cells expressing OCT4 was higher when serum-free IVP conditions were used, particularly when the oocyte culture medium was supplemented with PGE2. Overall, our results suggest that the experimental serum-free IVP conditions, and particularly the PGE2 enrichment of oocyte culture media, might interfere with regulation of the epigenetic reprogramming process that occurs during early development.

Few differentially expressed genes were revealed when comparing the ICM transcriptomes from both experimental serum-free IVP conditions. Five genes (H4C7, ADAM22, GUCY2C, SYNJ2, and ANKRD13B) were overexpressed and three (CPA4, GPR50, RGS3) were under-expressed in ICM when PGE2 was added to the oocyte culture medium. H4C7, which codes for H4 clustered Histone 7, and ADAM22 which encodes a member of the ADAM family (a disintegrin and metalloprotease domain), had previously been reported to be linked to regulating pluripotency and cell fate determination (Ren et al., 2014; Sun and Qi, 2014). GUCY2C, which encodes the guanylate cyclase C receptor, is known to reduce stem cell vulnerability to environmental damage by modulating the endoplasmic reticulum stress response (Kraft et al., 2017). SYNJ2 (Synaptojanin 2) has been identified as a player in epidermal growth factor receptor (EGFR) recycling and EGF-dependent migration (Csolle et al., 2020). ANKRD13B codes for a member of the Ankyrin repeat domain-containing protein 13 family and has recently been described as interfering with regulation of the endosome-to-lysosome trafficking of Caveolin 1 (Cav-1) (Burana et al., 2016). Intriguingly, the CAV1 gene was found to be overexpressed in ICM derived from PGE2-supplemented serum-free IVP when compared to the ICM transcriptome obtained under serum-supplemented conditions. Like SYNJ2, ANKRD13B also plays a role in regulating EGFR turnover (Mattioni et al., 2020). Among the under-expressed genes, the RGS3 gene that codes for a GTPase-activating protein was of interest; it had previously been described as an apoptosis inducer when overexpressed (Jiang et al., 2019). In addition, RGS3 may interact with Ephrin receptors to affect cell proliferation and differentiation (Geng et al., 2018). Our findings therefore highlighted candidate genes whose expression in ICM was specifically affected by the PGE2 enrichment of oocyte culture media during the periconceptional period.

Overall, PCR quantification of expression levels confirmed differences in gene expression we observed by RNA sequencing between the various IVP conditions. HERPUD1 displayed a significantly lower transcript expression in “SF” compared with the “SC” IVP treatment group by RNAseq but this differential expression was not confirmed by qPCR. For DNMT1, RNAseq analyses revealed a significantly higher expression in the “SF + PGE2” group compared with “SC” IVP treatment group but qPCR quantification indicated a trend toward a difference. Discrepancy between the two methods of gene expression quantification may be a consequence of gene structure such as size or exons number (Everaert et al., 2017). Nevertheless, about 83% of differential expression levels were consistent between RNAseq and real-time PCR, a percentage in keeping with previous reports.

Our experimental approach combining transcriptomic analysis and the evaluation of cryotolerance offered a convergent strategy to study the impact of IVP culture conditions on the different biological processes that occur specifically within the ICM during establishment of the first cell lineages. Although the cryotolerance properties of blastocysts are important criteria to evaluate the biological disturbances induced by laboratory culture techniques, the safety of IVP systems should ultimately be defined by the rate of pregnancies reaching full term.

## Data Availability Statement

The data presented in the study are deposited in the Gene Expression Omnibus data repository (https://www.ncbi.nlm.nih.gov/geo/), accession number GSE169593.

## Ethics Statement

The animal study was reviewed and approved by the animals were managed in accordance with the European Community Directive 2010/63/EU and under the license granted by the National Research Institute for Agriculture, Food and the Environment (INRA-UCEA). All experiments were carried out at the INRA experimental farm (registered as N°FRTB910 in the national registry for experimental farms). All protocols were approved by the local animal care and use committee and, for the later collection periods, by the local ethics committee (Registered as N°12/086 in the National Ethics Committee registry).

## Author Contributions

FN, GC, and BM-L designed the experiments and evaluated the results. FN and GC prepared the figures and contributed to compiling the manuscript. All the authors performed the experiments and approved the version submitted.

## Conflict of Interest

The authors declare that the research was conducted in the absence of any commercial or financial relationships that could be construed as a potential conflict of interest.

## Footnotes

1 https://doi.org/10.15454/1.5572348210007727E12

2 https://imagej.nih.gov/ij/

3 http://genomeast.igbmc.fr/

4 https://www.ncbi.nlm.nih.gov/tools/primer-blast/

5 https://bioconductor.org/help/course-materials/2017/CSAMA/labs/2-tuesday/lab-03-rnaseq/rnaseqGene_CSAMA2017.html

6 https://www.ensembl.org/index.html

7 http://pantherdb.org
