# Supplementary material for: PGE2 Supplementation of Oocyte Culture Media Improves the Developmental and Cryotolerance Performance of Bovine Blastocysts Derived From a Serum-Free in vitro Production System, Mirroring the Inner Cell Mass Transcriptome
Source: Front Cell Dev Biol. 2021 Jun 7;9:672948. doi: 10.3389/fcell.2021.672948 (PMC8215579; doi:10.3389/fcell.2021.672948)
Supplement: Supplementary file 1 [file Table_1.docx]

| gene name | accession number | primer forward (5'-->3') | primer reverse (5'-->3') | amplicon size |
| --- | --- | --- | --- | --- |
| JARID2 | ENSBTAG00000012938 | CAGGAGAACGAGAACGTGGT | AGTTCTCAATGCTGCCGTTT | 172 |
| DNMT1 | ENSBTAG00000002736 | AAGCTGACCATGGACCTGAC | CTTTTTCTTCCGCTTCATGC | 231 |
| DDIT3 | ENSBTAG00000031544 | ACGGCTTCTTCAGCACAACT | CCGGTGCTTGTCTAGGATGT | 164 |
| MYBBP1A | ENSBTAG00000007430 | CAGGCATTCTGCTGTGAGAA | TCCCGGCAAAAACAAATAAG | 212 |
| HERPUD1 | ENSBTAG00000016896 | TGGAAGCCTGGTATGAGGAC | GGGAGGTGTGTGTGACCTCT | 177 |
| GADD45A | ENSBTAG00000013860 | CACCAAGTGCGCAAGAGATA | GGCTGAGTAGGTCCAATCCA | 221 |

Table SMM1: Primer pairs for quantitative PCR reactions
