# Supplementary material for: PGE2 Supplementation of Oocyte Culture Media Improves the Developmental and Cryotolerance Performance of Bovine Blastocysts Derived From a Serum-Free in vitro Production System, Mirroring the Inner Cell Mass Transcriptome
Source: Front Cell Dev Biol. 2021 Jun 7;9:672948. doi: 10.3389/fcell.2021.672948 (PMC8215579; doi:10.3389/fcell.2021.672948)
Supplement: Supplementary file 2 [file Table_2.docx]

**Table SR2.** Differentially expressed genes in ICM between « SC » and « SF » IVP treatment groups

|  | pvalue <0.05 | padjusted <0.05 |
| --- | --- | --- |
| underexpressed in « SC » | 1302 | 382 |
| overexpressed in « SC » | 1274 | 316 |
| total DEGs | 2576 | 698 |
