# Supplementary material for: PGE2 Supplementation of Oocyte Culture Media Improves the Developmental and Cryotolerance Performance of Bovine Blastocysts Derived From a Serum-Free in vitro Production System, Mirroring the Inner Cell Mass Transcriptome
Source: Front Cell Dev Biol. 2021 Jun 7;9:672948. doi: 10.3389/fcell.2021.672948 (PMC8215579; doi:10.3389/fcell.2021.672948)
Supplement: Supplementary file 4 [file Table_4.docx]

**Table SR4.** Differentially expressed genes in ICM between « SF+PGE2 » and « SF » IVP treatment groups

|  | pvalue <0.05 | padjusted <0.05 |
| --- | --- | --- |
| underexpressed in « SF+PGE2 » | 382 | 3 |
| overexpressed in « SF+PGE2 » | 470 | 5 |
| total DEGs | 852 | 8 |
