# Supplementary figures and images for: PGE2 Supplementation of Oocyte Culture Media Improves the Developmental and Cryotolerance Performance of Bovine Blastocysts Derived From a Serum-Free in vitro Production System, Mirroring the Inner Cell Mass Transcriptome
Source: Front Cell Dev Biol. 2021 Jun 7;9:672948. doi: 10.3389/fcell.2021.672948 (PMC8215579; doi:10.3389/fcell.2021.672948)

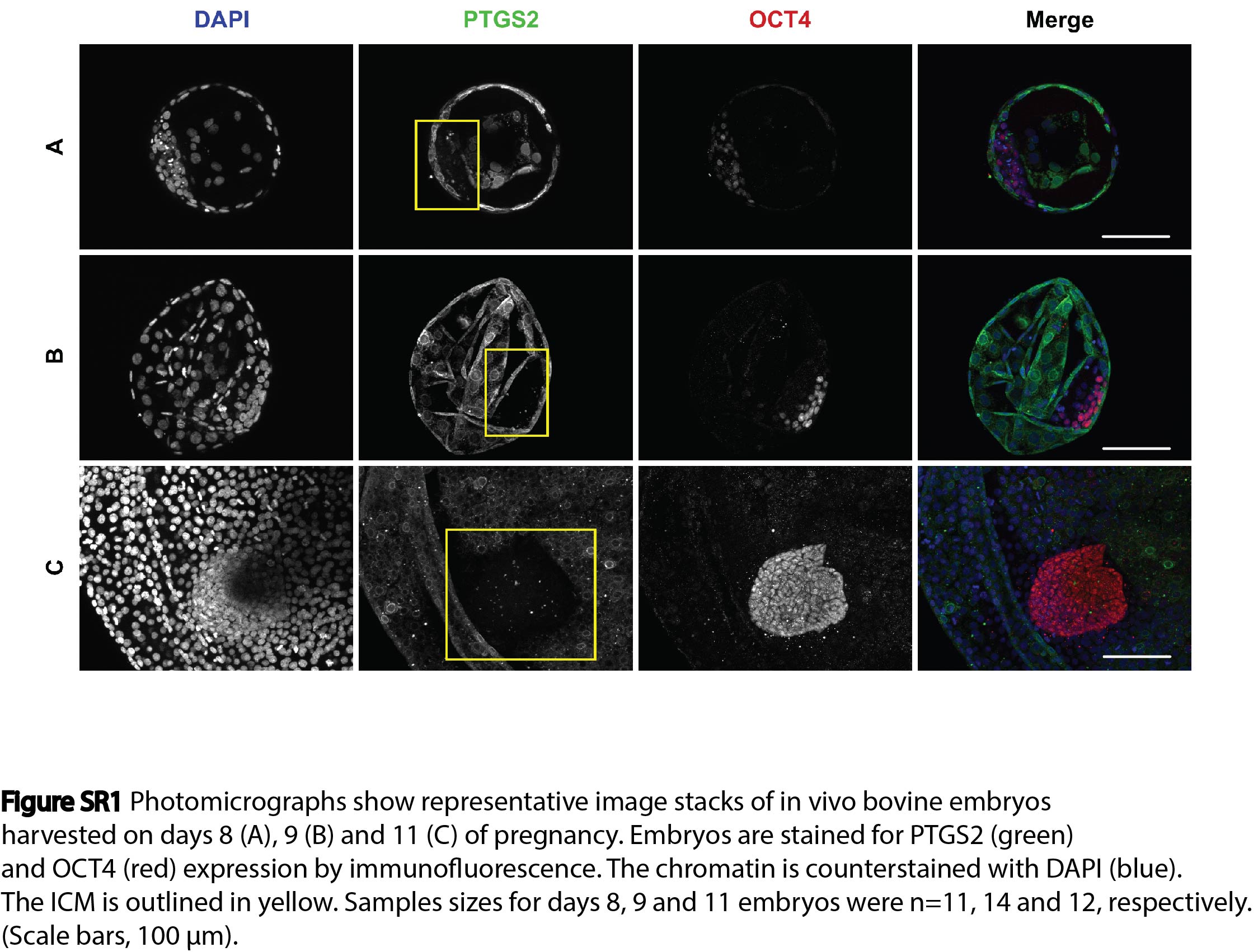

Supplement: Supplementary file 7 [file Image_1.JPEG]

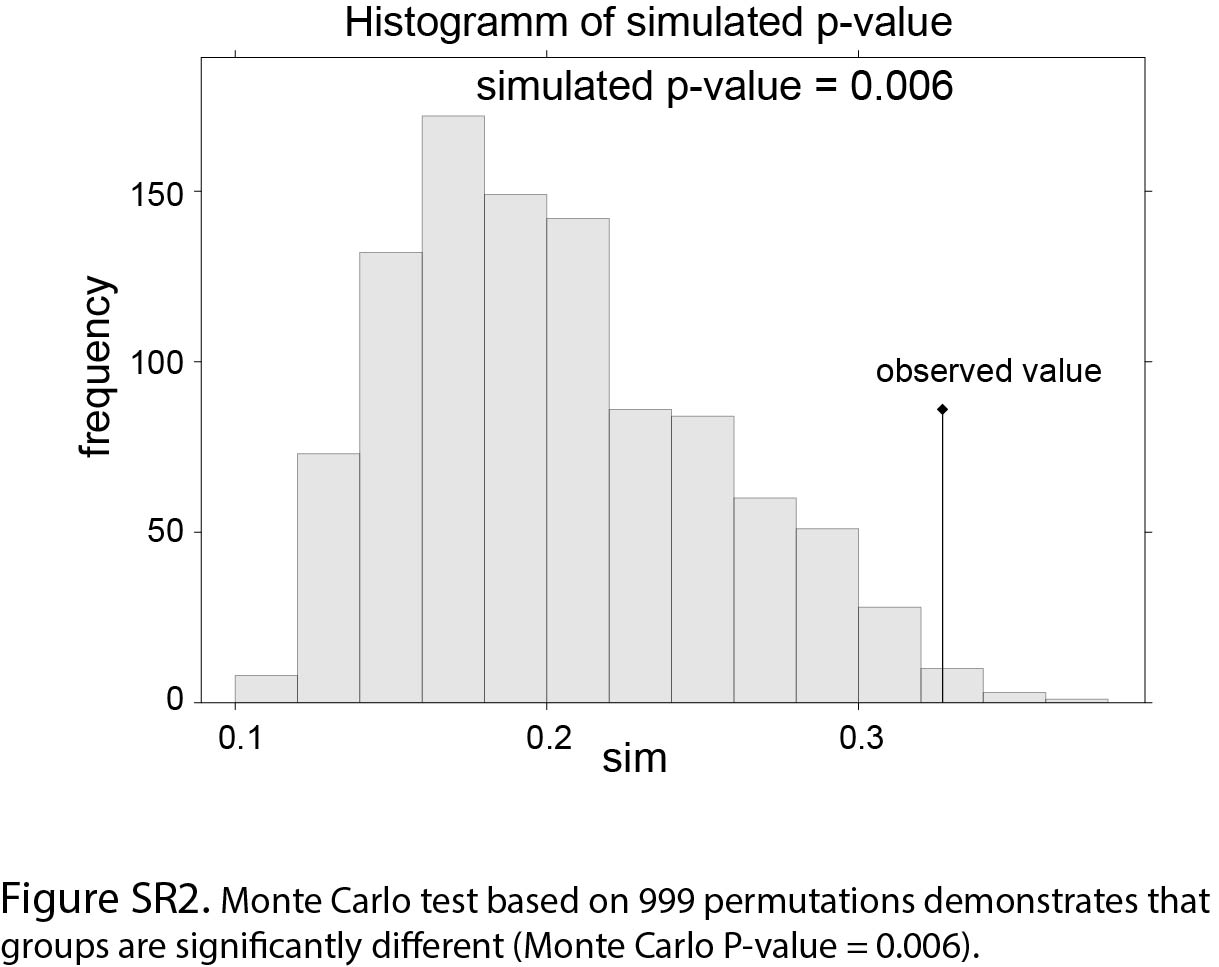

Supplement: Supplementary file 8 [file Image_2.JPEG]
